# An integrated clinical program and crowdsourcing strategy for genomic sequencing and Mendelian disease gene discovery

**DOI:** 10.1038/s41525-018-0060-9

**Published:** 2018-08-13

**Authors:** Alireza Haghighi, Joel B. Krier, Agnes Toth-Petroczy, Christopher A. Cassa, Natasha Y. Frank, Nikkola Carmichael, Elizabeth Fieg, Andrew Bjonnes, Anwoy Mohanty, Lauren C. Briere, Sharyn Lincoln, Stephanie Lucia, Vandana A. Gupta, Onuralp Söylemez, Sheila Sutti, Kameron Kooshesh, Haiyan Qiu, Christopher J. Fay, Victoria Perroni, Jamie Valerius, Meredith Hanna, Alexander Frank, Jodie Ouahed, Scott B. Snapper, Angeliki Pantazi, Sameer S. Chopra, Ignaty Leshchiner, Nathan O. Stitziel, Anna Feldweg, Michael Mannstadt, Joseph Loscalzo, David A. Sweetser, Eric Liao, Joan M. Stoler, Catherine B. Nowak, Pedro A. Sanchez-Lara, Ophir D. Klein, Hazel Perry, Nikolaos A. Patsopoulos, Soumya Raychaudhuri, Wolfram Goessling, Robert C. Green, Christine E. Seidman, Calum A. MacRae, Shamil R. Sunyaev, Richard L. Maas, Dana Vuzman, Undiagnosed Diseases Network, Brigham and Women’s Hospital FaceBase Project, Brigham Genomic Medicine (BGM)

**Affiliations:** 1 Division of Genetics, Department of Medicine, Brigham and Women’s Hospital, Harvard Medical School, Boston, MA 02115 USA; 2 Division of Cardiovascular Medicine, Department of Medicine, Brigham and Women’s Hospital, Harvard Medical School, Boston, MA 02115 USA; 3 Department of Genetics, Harvard Medical School, Boston, MA 02115 USA; 4 Howard Hughes Medical Institute, Brigham and Womens Hospital, Boston, MA 02115 USA; 5 Broad Institute of Harvard and MIT, Cambridge, MA 02142 USA; 6 Division of Medical Genetics and Metabolism, Department of Pediatrics, Massachusetts General Hospital, Harvard Medical School, Boston, MA 02114 USA; 7 Division of Genetics and Genomics, Department of Medicine, Boston Childrens Hospital, Harvard Medical School, Boston, MA 02115 USA; 8 Division of Gastroenterology, Hepatology and Nutrition, Department of Medicine, Boston Childrens Hospital, Harvard Medical School, Boston, MA 02115 USA; 9 Division of Gastroenterology, Hepatology and Endoscopy, Department of Medicine, Brigham and Womens Hospital, Harvard Medical School, Boston, MA 02115 USA; 10 Dana-Farber Cancer Institute, Boston, MA 02115 USA; 11 Cardiovascular Division, Department of Medicine; Department of Genetics; McDonnell Genome Institute, Washington University School of Medicine, St Louis, MO 63110 USA; 12 Department of Medicine, Brigham and Womens Hospital, Harvard Medical School, Boston, MA 02215 USA; 13 Endocrine Unit, Massachusetts General Hospital and Harvard Medical School, Harvard Medical School, Boston, MA 02114 USA; 14 Division of Plastic and Reconstructive Surgery, Department of Surgery, Massachusetts General Hospital, Harvard Medical School, Boston, MA 02114 USA; 15 Feingold Center for Children, Childrens Hospital Boston at Waltham, Waltham, MA 02453 USA; 16 Department of Pediatrics, Cedars-Sinai Medical Center, David Geffen School of Medicine at UCLA, Los Angeles, CA 90048 USA; 17 Department of Orofacial Sciences, University of California San Francisco, San Francisco, CA 94143 USA; 18 Department of Neurology, Brigham and Womens Hospital, Harvard Medical School, Boston, MA 02115 USA; 19 Division of Rheumatology, Allergy and Immunology, Department of Medicine, Brigham and Womens Hospital and Harvard Medical School, Boston, MA 02115 USA; 20 Institute of Inflammation and Repair, University of Manchester, Manchester, UK

## Abstract

Despite major progress in defining the genetic basis of Mendelian disorders, the molecular etiology of many cases remains unknown. Patients with these undiagnosed disorders often have complex presentations and require treatment by multiple health care specialists. Here, we describe an integrated clinical diagnostic and research program using whole-exome and whole-genome sequencing (WES/WGS) for Mendelian disease gene discovery. This program employs specific case ascertainment parameters, a WES/WGS computational analysis pipeline that is optimized for Mendelian disease gene discovery with variant callers tuned to specific inheritance modes, an interdisciplinary crowdsourcing strategy for genomic sequence analysis, matchmaking for additional cases, and integration of the findings regarding gene causality with the clinical management plan. The interdisciplinary gene discovery team includes clinical, computational, and experimental biomedical specialists who interact to identify the genetic etiology of the disease, and when so warranted, to devise improved or novel treatments for affected patients. This program effectively integrates the clinical and research missions of an academic medical center and affords both diagnostic and therapeutic options for patients suffering from genetic disease. It may therefore be germane to other academic medical institutions engaged in implementing genomic medicine programs.

## Introduction

Although rare as unique disease entities, Mendelian disorders are altogether more frequent than previously thought and collectively affect millions of patients worldwide.^1^ Often life-threatening or chronically debilitating, Mendelian diseases are estimated to account for 12 and 2% of pediatric and adult hospital admissions, respectively.^2^ Early molecular diagnosis and individualized management that targets the underlying pathophysiology can improve the quality of life for many of these patients. There are approximately 7,000 clinically described rare Mendelian diseases, and only about half of these have a clearly described genetic cause.^2^ The total number of Mendelian diseases may be substantially higher,^3^ leaving many new conditions to be clinically described. In addition, the benefits of genomic sequencing in Mendelian diseases extend beyond individuals with rare disorders. Genes responsible for large effects in Mendelian disorders may contribute moderate effects to more common forms of the same or related disease phenotypes.^4–6^

## Program overview

**Fig. 1:**
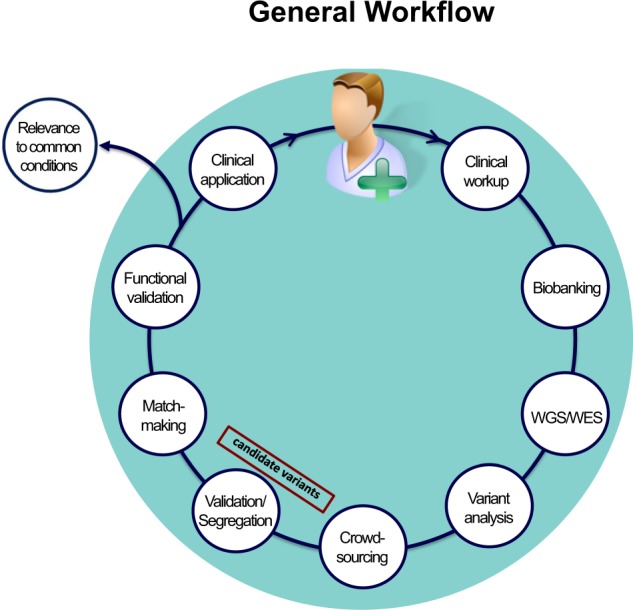
Workflow overview. The workflow begins with the clinical assessment of cases, where referring physicians present cases of presumptive unknown monogenic etiology from their clinical practice for the collective development of a case solution strategy. Based on the inferred inheritance mode, the most informative family members are selected for genomic sequencing followed by analysis of the WES/WGS data using a computational pipeline designed to identify rare Mendelian variants. A final candidate gene list is prioritized using in-house bioinformatic tools, literature surveys, and crowdsourcing. The final candidate gene is confirmed by segregation analysis, matchmaking for second case hits, and by in vitro and in vivo functional studies. Both genomics and functional biology thus inform the diagnosis and clinical management of individual patients, while unique patient conditions provide insight into gene and pathway function

We describe a multidisciplinary integrated clinical diagnostic program situated in an academic medical center whose goal is to identify the genetic basis of presumptive undiagnosed Mendelian conditions. This differs from typical clinical diagnostic services, which primarily test for genetic variants whose association with clinical disorders is often supported by substantial prior evidence. The central premise that informs this multidisciplinary integrated model is that a particular subset of clinical genetics cases that are encountered in the hospital and outpatient setting offer unique opportunities for gene discovery and disease pathway elucidation and, in some cases, for the re-purposing of existing therapeutics or the advent of novel therapeutic and clinical management options (Fig. 1).

At the present, several national sequencing projects aim to establish platforms for genomic epidemiology that include a health database and biobank. Most notably, these include the US “All of Us” Research Program (https://allofus.nih.gov), the Million Veterans Program (MVP) (https://www.research.va.gov/mvp/), the UK 100,000 Genomes Project (https://www.genomicsengland.co.uk), The Genome of the Netherlands,^7^ and Korean Genome and Epidemiology Study (KoGES).^8^ In addition, several smaller programs with a more precise focus on monogenic diseases, such as the National Institutes of Health (NIH) Undiagnosed Diseases Network (UDN),^9^ the Idiopathic Diseases of Man (IDIOM) study,^10^ the Centers for Mendelian Genomics (CMG),^11,12^ and the UK Deciphering Developmental Disorders (DDD),^13^ feature the application of genome sequencing integrated with clinical assessment and multidisciplinary gene discovery.

Here, we describe a multidisciplinary integrative clinical and research program called Brigham Genomic Medicine (BGM) for patients presenting with a wide range of phenotypic traits and family history consistent with a Mendelian pattern of inheritance. A distinctive feature of BGM is its focus on undiagnosed monogenic disease cases that are refractory to standard diagnostic approaches. In addition, the program provides an integrated and continuous pathway from case ascertainment to treatment. Whenever reasonable, a new or improved management strategy is initiated for the patients based on novel genomic findings.

The main objectives of the program described here are to: (1) use state-of-the-art clinical and research methods to discover the genetic causes of undiagnosed Mendelian cases; (2) provide new diagnostic and treatment options for these patients; and (3) promote the clinical implementation of genomic medicine by educating physicians and researchers to recognize possible genetic disorders. An important feature of this program and its accompanying team is its interdisciplinary nature. For example, in addition to academic physicians from different clinical specialties and subspecialties, the program includes clinical molecular geneticists, genetic counselors, and scientists with expertise in statistical genetics, genomic analysis, bioinformatics, molecular genetics, and experimental disease modeling. The interdisciplinary team is therefore well-equipped to tackle analytical and diagnostic challenges as they arise.

The program team routinely includes about 25 members who meet weekly to review 1–3 cases. Meetings are typically structured around new case presentations or discussion of ongoing analyses for existing cases. New cases are presented by referring physicians with the goal of discussing the medical appropriateness of the case, and the a priori likelihood of a tractable solution via whole-exome and whole-genome sequencing (WES/WGS); a decision is then made about each case’s acceptance to the program. Decisions require a quorum of at least three clinicians and three biomedical scientists. If a case is accepted for WES/WGS, the patient and relevant family members are consented to BGM research protocol, samples are sent to a commercial sequencing provider, and the resulting BAM sequence files are analyzed further in-house using a genomic sequence analysis pipeline, as described below. The results of the in-house analysis pipeline are then curated and compared to the “clinical genomic sequencing” report, when available.

Once the variant list has been generated, the referring physician and program team reconvene to discuss and refine the results. These meetings are typically preceded by a distribution of the case synopsis and variant list via a crowdsourcing portal, also described below. To accommodate busy clinical work schedules, referring physicians may participate either in person or via conference call. To encourage the participation of clinical faculty and physicians, the weekly case conference provides continuing medical education (CME) credit to clinical participants.

## Case ascertainment and informed consent

To optimize the probability of solving a case, specific criteria are used to select cases and to determine their suitability for WES or WGS. These selection criteria include: (1) the likelihood of solving the case, which in turn is a function of the likelihood that a phenotype has an underlying monogenic etiology, the predicted inheritance mode, and the availability of the family members needed to solve the case; (2) the disease morbidity that might be alleviated if the case were solved; (3) the potential for scientific discovery (i.e., if we suspect a novel pathway is involved, we are more inclined to accept the case); and (4) the potential relevance of the case to more common diseases with similar phenotypes.

Clinicians from all clinical departments are encouraged to refer Mendelian cases that could have broad impact on patient care if the causal gene were identified. To date, physician champions in the departments of genetics, pediatrics newborn medicine, gastroenterology, cardiology, rheumatology, endocrinology, nephrology, dermatology, and hematology as well as the NIH UDN, and the National Institute of Dental and Craniofacial Research (NIDCR) FaceBase Consortium have referred most of the cases to the program. However, since the ultimate impact of solving a case cannot be determined in advance, all cases involving medically significant disease are potentially eligible for consideration. A genetic counselor and a clinical geneticist examine the referred patients and review all available medical records and the family history. Patients who appear to be good candidates for WES or WGS have their cases presented by their referring physician to the program, as described above, to determine whether they meet the inclusion criteria and to define the optimal testing strategy; for example, whether to use WES, WGS, or some intermediate approach such as chromosomal microarray. WES is ordinarily the default sequencing strategy, primarily for cost considerations, but factors favoring WGS include: (1) cases in which a chromosomal rearrangement, insertion or deletion, or copy number variation is suspected; (2) cases in which uniform genome coverage is preferred; and (3) cases in which a regulatory variant is assumed due to high suspicion of a specific monogenic etiology despite a negative WES. The cases selected for WES/WGS are described using Human Phenotype Ontology (HPO), which provides a structured, comprehensive set of terms for the phenotypic abnormalities characterizing human disease.^14–16^

Unlike many other similar programs that may accept undiagnosed cases at large, the cases selected for WES/WGS analysis via this program are unique in that in general they are not routinely solvable by conventional WES of the proband alone or by application of conventional clinical or commercial WES analytical pipelines. Thus, patients and family members undergo an informed consent process to participate in a research study and to have their data shared for a crowdsourcing analysis in accordance with an institutional review board (IRB)-approved protocol. To protect privacy, each patient is assigned an anonymous identification number at admission.

## Genomic sequencing and data analysis

Blood is drawn and DNA is extracted for WGS, or a combination of WES (to detect coding variants) and single-nucleotide polymorphism chromosomal microarrays to detect structural and copy number variants, and to conduct genome-wide linkage analysis if necessary. Sequencing is performed in a CLIA (Clinical Laboratory Improvement Amendments)-certified laboratory when clinical indications for sequencing exist and once reimbursement by insurers is approved; otherwise, sequencing is conducted in a research laboratory.

Our preferred sequencing and analytical approach utilizes specific inheritance models for each case in addition to the following assumptions to identify a small list of candidate variants without bias from known biological or phenotypic gene associations: (1) rare, severe, monogenic disease is typically caused by coding variants that are absent or extremely rare in the general population; (2) rare, severe monogenic diseases are caused by highly penetrant genetic variants. For example, in cases with only a single affected family member for which dominant de novo and recessive inheritance modes are each possible, our preferred approach is to sequence the trio consisting of proband and both parents and to analyze the proband’s variants with respect to the parental genotypes. In such a case, we expect to identify 1–2 de novo variants, 0–1 homozygous variants, and 2–5 compound heterozygous variants at allele frequencies lower than 0.1% in the population. In consanguineous recessive cases and/or recessive cases with available affected siblings, the DNA of the proband can be sequenced along with that of an affected sibling, and the shared recessive variants can then be further tested for appropriate segregation among unaffected siblings by simple PCR analysis or Sanger sequencing. In dominant cases with multiple affected family members, we prefer to sequence the proband in parallel with the *most remote* affected available relative, most commonly a cousin, to reduce the number of candidate variants to the smallest number possible, ideally less than 10. Finally, individual cases without available relatives or cases where the phenotype requires both a discrete environmental stimulus and a genetic susceptibility variant can occasionally be solved from a single genome sequence.

**Fig. 2:**
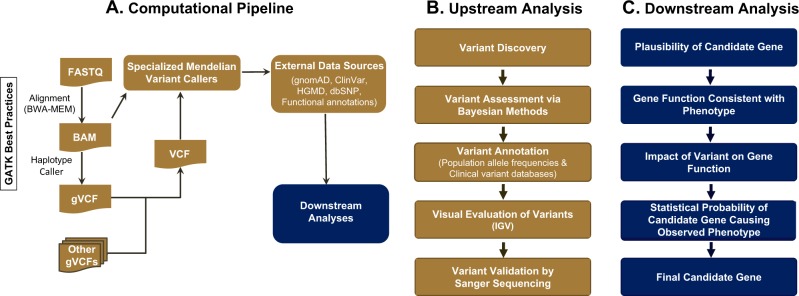
Overview of the analytical pipeline. **a** The computational pipeline is shown, including discrete steps for the alignment of read data, joint genotyping, variant prioritization programs, incorporation of external data sources, and variant visualization, culminating in downstream analyses; **b** detailed breakdown of the upstream analysis is shown, including the production of validated, annotated, and prioritized candidate variants; **c** detailed breakdown of the downstream analysis culminates in the evaluation of candidate genes and the variants within them for causality for the condition of interest

The two major components of the analytical pipeline include: (1) alignment and calling of sequence variants from WES/WGS data, and (2) interpretation of the functional consequences of the identified sequence variants (Fig. 2a). This pipeline includes algorithms that improve the sensitivity and specificity of Mendelian calling using probabilistic models that capitalize on data related to the mode of inheritance, and tools to identify non-coding regulatory variants in WGS data (Fig. 2b). One of the distinguishing features of our WES/WGS computational analysis pipeline is its optimization for Mendelian gene discovery with callers tuned to specific inheritance modes (e.g., de novo dominant, shared dominant, or other inheritance modes). This innovation substantially reduces the number of candidate variants that require confirmatory Sanger sequencing at additional time and cost. These callers are optimized for high specificity and sensitivity by using familial information and unrelated samples to detect technical artifacts present in the sequencing and alignment process. These callers also extend the power of joint calling by gauging the presence of technical artifacts along with estimation of the prevalence of an allele at any variant call location. Independent Sanger sequencing has confirmed the high accuracy of these callers (Mohanty et al., submitted). The unified Bayesian framework they employ enables the best decision about whether an allele is present or if alternate reads are present at the location as a technical artifact. Downstream analysis calculates the statistical probability of detecting each variant by chance based on a constraint missense *Z* score, and the probability of being loss-of-function intolerant (pLI)^17^ (Fig. 2c). In addition, we use Phenomizer,^18,19^ a diagnostics tool that uses semantic similarity metrics to measure phenotypic similarity between queries and hereditary diseases annotated with the use of the HPO. BGM also uses this pipeline to analyze cases from the National Human Genome Research Institute (NHGRI) UDN^9^ and from the NIDCR FaceBase Consortium.^20,21^

Putative causal variants must fulfill the following criteria: (1) co-segregation with the disease based on the inheritance mode; (2) rare with a minor allele frequency of 0.1% or less in all populations from unaffected consortia (e.g., gnomAD (genome Aggregation Database) and (3) predicted functional impact on the gene product.

## Causality of candidate genes

To establish the causality of variants identified by WES/WGS for a given case, appropriate follow-up studies are tailored to address the relevance of the gene and genetic variant to the disease phenotype in question, in collaboration with faculty across the academic medical center. This approach makes use of a flexible two-part strategy: (1) identifying additional cases (e.g., two or more independent cases) with a similar phenotype bearing presumptive functional variants in the same gene, typically via Matchmaker Exchange;^22^ and (2) empiric functional experiments. For the latter, we use a wide range of experimental systems for biological validation of the top 1–3 identified candidate genes, including: (a) in vitro protein and biochemical assays to study the interactive and enzymatic effects of specific variants on protein function; (b) use of cellular models, including human induced pluripotent stem cells and CRISPR (Clustered Regularly Interspaced Short Palindromic Repeats) to investigate the effects of gene inactivation or specific variants on cellular function; and (c) use of engineered zebrafish and mouse models to recapitulate human phenotypes.

As an example (Table 1, case 7), we identified distinct PIEZO2 variants (p.Glu2727del and p.Ile802Phe) in two separate patients with the musculoskeletal contracture and respiratory disease Distal Arthrogryposis type 5 (DA5).^23^ One of the patients presented to our emergency department in acute respiratory distress during flu season. Electrophysiological studies performed with a collaborator demonstrated that the identified PIEZO2 variants affect biophysical properties related to channel inactivation and caused PIEZO2-dependent, mechanically activated currents to recover faster from inactivation, resulting in increased channel activity in response to a mechanical stimulus. These findings indicated that DA5 is caused by gain-of-function variants in *PIEZO2*.

In sum, we pursue biological validation through collaboration with investigators with expertise in functional assays relevant to the discovered genes. Thus, whenever required, we seek to establish collaborations with scientists external or internal to our institution, whose expertise focuses on the gene or disease of interest.

**Table 1:** Brigham Genomic Medicine (BGM) genetic diagnoses to date

BGM no.	Gene	Inheritance	Diagnosis/novel or known syndrome, if known–phenotype exp.?	Prior genetic testing	Sequencing strategy	Solution strategy	Case source	Ref. or status
1	*CHST11*	AR	Skeletal malformation, malignant lymphoproliferative disease/novel	MA, K	Pbd WGS; S	CS	BWH, genetics	^31^
2	*WISP3*	AR (cons.)	Prog. pseudorheumatoid arthropathy, childhood (PPAC)/ OMIM 208230	Unknown	Pbd WGS; S	CS	BWH, Rheum	^30^
3	*LOX*	AD	Thoracic aortic aneurysm and dissection (TAAD)/novel	Panel	Pbd, c WGS; S	CC, MC, MO	BWH, Cardio	^29^
4	*DOCK8*	AR (cons.)	Infantile inflammatory bowel disease/novel	WES	Pbd WES re-anly	CC	BCH, GI	In prep.
5	*C3*	AD	Lower extremity ischemia/novel	None	Pbd, c WGS, S	CS, MC, FE	BWH, Rheum	In prep.
6	*MVK*	AR	Infantile-onset Crohn’s disease	None	Pbd, M WES	CC BCH, GI		In prep.
7	*PIEZO2*	AD (de novo)	Distal arthrogryposis type 5/gene discovery for OMIM 108145	None	Trio WGS	CC, MC, FE	BWH, genetics	^23^
8	*TRPM4*	AD	Right-sided structural heart defects and conduction defects	None	WES Pbd, c, M, F	CC	BCH, genetics	^38^
9	*OBSCN/TTN* ^a^	AR	Nemaline myopathy	None	Trio WES	CC	BCH, genetics	^38^
10	*TTN / GJB2* ^a^	AR	Centronuclear myopathy and bilateral sensorineural hearing loss	None	Trio WES	CC	BCH, genetics	^38^
11	*CTLA4*	AD	Multisystem autoimmune disorder/novel	WES	WES s, M, 2F	CC	BWH, GI	^39^
12	*CAPZB*	AD (de novo)	Craniofacial anomalies and hypotonia/novel	K	Pbd WGS	CC, MO	FB/DGAP	^40^
13	*RSPRY1*	AR (cons.)	Progressive spondyloepimetaphyseal dysplasia and intel. disability/novel	None	Pbd WES	CC, MC, MO	FB/KFSH	^41^
14	*MAP3K7*	AD (de novo)	Global developmental delay and dysmorphic facies	WES	Trio WGS	CS, MC	FB/FCC	^42, 43^
15	*F12*	AD	Angioedema type 3/OMIM 610618	None	Pbd WGS	CC	BWH, Allergy & Imm.	^42^
16	*POLR1A*	AD (de novo)	Acrofronto-facionasal dysostosis/OMIM 616462	None	Trio WES	CS	FB/CHLA	^d^
17	*NFIX*	AD (de novo)	Marshall–Smith syndrome+glioma/OMIM 602535+Pheno. exp.	None	Trio WES	CS	FB/CHLA	^d^
18	*FBN2*	AD (de novo)	Congenital contractural arachnodactyly/OMIM 121050	None	Trio WES	CS	FB/CHLA	^d^
19	*DCHS1*	AR	Van Maldergem syndrome 1 / OMIM 601390+Phen Exp.	K, Panel	Trio WES	CS	FB/BCH	^d^
20	*ALX1*	AR	Frontonasal dysplasia-microphthalmia-facial clefting/OMIM 613456	None	Trio WES	CS	FB/MGH	In prep.
21	*CDH1*	AD	Blepharocheilodontic Syndrome 1/OMIM 119580	None	Pbd, M WES	CC, MC	FB/CHLA	^d^
22	*CNTNAP1*	AR	Lethal congenital contracture syndrome 7/OMIM 616286	MA	Quad WES	CC	HUDN/MGH	FE
23	*MAGEL2*	AD (de novo)	Schaaf–Yang syndrome/OMIM 614547	MA, K	Trio WES	CS	HUDN/BCH	Closed
24	*PAX6* ^b^	AD (de novo)	Aniridia plus clubfoot, craniofacial disorder/OMIM 106210+Pheno. exp.	Micro	Trio WES	CS	FB/CHLA	^d^
25	*EPCAM*	AR	Pancreatic insufficiency, global developmental delay/OMIM 613217	Panel	Trio WES	CS	HUDN/BCH	Closed
26	*SNTA1*	AD	Isolated hypoparathyroidism/novel	None	Pbd, c WES	CS	MGH/Endocrin	FE
27	*TRPV4*	AR	Distal hereditary motor neuropathy/OMIM 606071+Pheno. Exp.	None	Trio WES	CC	BCH/MCODR	FE
28	*ITGB2* ^c^	AD	Purpura fulminans/novel	None	Cohort WES	CS, MC, FE	UIHC	FE
29	*MED12* ^b^	XLR	Congenital anomalies, polydactyly/some similarity to OMIM 300895	MA, FISH	Trio WGS	CS	HUDN/BCH	FE
30	*MED13L*	AD (de novo)	Pierre Robin syndrome, ASD/OMIM 616789	MA, FISH, K	Trio WES	CS	FB/UCSF	https://www.facebase.org/ ^d^

Note that for HUDN cases, only cases solved independently by BGM are listed. Mutational data and case details are available upon request

*AR* autosomal recessive, *AD* autosomal dominant, *cons* consanguineous pedigree, *MA* microarray, *K* karyotype, *Panel* gene panel and/or individual gene tests, *FISH* fluorescence in situ hybridization, *WES* whole-exome sequencing, *WGS* whole-genome sequencing, *Pbd* proband, *c* cousin(s), *s* sib(s), *re-anly* re-analysis, *M* mother, *F* father, *S* segregation analysis, *Trio* proband M, F, *CS* crowdsourcing, *CC* case champion, *MC* multiple cases, *MO* model organism, *FE* functional experiments (completed or ongoing), *BWH* Brigham and Women’s Hospital, *BCH* Boston Children’s Hospital, *MGH* Massachusetts General Hospital, *DGAP* Developmental Genome Anatomy Project, *FB* NIDCR FaceBase Consortium, *KFSH* King Faisal Specialist Hospital and Research Center, *FCC* Feingold Center for Children, *CHLA* Children’s Hospital Los Angeles, *HUDN* Harvard Undiagnosed Disease Network-Clinical Site, *UIHC* University of Iowa Hospitals and Clinics, *UHF* University of Florida Health, *MCODR* Manton Center for Orphan Disease Research, *Rheum* rheumatology, *Cardio* cardiovascular medicine, *GI* gastroenterology, *Allergy & Imm* allergy and immunology, *Endocrin* endocrinology

^a^Possible digenic inheritance, based on phenotype

^b^May not explain entire phenotype

^c^Potential synthetic enhancement of phenotype due to genetic interaction with additional loci within in the same functional pathway

^d^De-identified genomic sequences and clinical data available via the NIDCR FaceBase Hub website (https://www.facebase.org/) contingent upon approval

## Crowdsourcing and matchmaker analyses

Solving undiagnosed and complex cases requires diverse knowledge and expertise. While many cases benefit from analysis via a “case champion” model in which one individual, usually with special knowledge of the disease or of a primary candidate gene, drives the analysis, this strategy is not always available or successful. To further help solve challenging cases we have implemented an innovative crowdsourcing strategy among faculty and staff to utilize specialized expertise across the entire academic medical center. Our crowdsourcing system is restricted to physicians, clinical staff, and research faculty of the parent institution. All cases are fully de-identified, allowing useful information to be shared without violating HIPAA (Health Insurance Portability and Accountability Act) regulations or patient privacy. The HIPAA-compliant portal is securely installed behind a firewall and integrated with the institution’s authentication system (i.e., username and password) to provide controlled access.

**Fig. 3:**
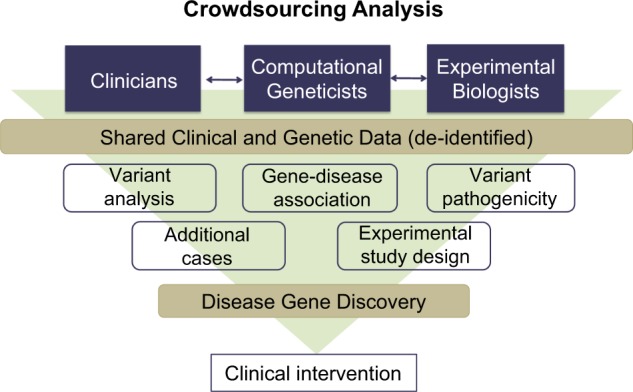
Crowdsourcing of Mendelian cases. Clinical and genomic data of cases under analysis are presented in fully de-identified format via a secure portal and in compliance with HIPAA and patient privacy regulations. This crowdsourcing mechanism provides clinicians, researchers, and data analysts with the opportunity to “interactively” analyze the data, vet analytical approaches, explore follow-up options, and to obtain second, third, or fourth opinions before finalizing a particular analytical and validation strategy. In some cases, the crowdsourcing strategy can serve as a matchmaker for a second case based on the phenotype, genotype, or both

The patient data shared for crowdsourcing include de-identified clinical and laboratory details as well as a list of variants identified by WES/WGS (Fig. 3). Together with BGM faculty and collaborators, the main bioinformatic analyst for each case reviews the variant and clinical data and organizes the analysis and presentation of the case. In addition, other scientists and clinicians throughout the institution can request to participate in the crowdsourcing analysis. These participating analysts work collaboratively or independently on cases within a timeframe specified by the program director, but with a degree of flexibility that encourages participation. In addition, there is no minimum time commitment for participation in the crowdsourcing analyses, and participating analysts spend from one to several hours a week. Once the analyses are codified, the main analyst summarizes the various analytical inputs and presents the case at the weekly program meeting to all program staff including those who participated in the crowdsourcing analysis. The interdisciplinary program team discusses the findings and decides on the next steps. This crowdsourcing innovation helps identify candidates and effective validation assays that otherwise would have been missed, engages the collective expertise of a broad range of faculty in the exercise of analyzing Mendelian disease cases, and also serves a broad educational purpose.

Our first 30 solved cases span a wide range of disorders presenting in children and adults and were drawn from multiple referral sources (Table 1). Criteria for establishing causality include: (1) the existence of multiple independent cases with similar phenotypes and variants in the same gene; (2) recapitulation of the human disease phenotype, in whole or in part, in an appropriate animal model, typically mouse or zebrafish; and (3) demonstration of a molecular defect in an appropriate cellular or biochemical assay.

Seventeen of the cases were solved using the crowdsourcing mechanism among ~50 participating faculty, scientists, physicians, genetic counselors, and fellows in our institution, while the remaining cases employed a case champion model. For example, one illustrative case that was resolved via the crowdsourcing mechanism involved a male infant with symptoms consistent with very early-onset inflammatory bowel disease (VEO-IBD) and hearing loss (Ouahed, Snapper et al. manuscript in preparation). The patient’s clinical and genomic data were posted on the program portal. A genetic counselor annotated the potential relevance of a novel gene variant with the comment that: “Variants in a homologous gene are associated with variable clinical presentations in patients with familial hemophagocytic lymphohistiocytosis (HLH) type 5 - sensorineural hearing deficit, abnormal bleeding, and, most frequently, severe diarrhea only present in early-onset disease infantile IBD and hearing loss.” In this instance, the annotation for the homologous gene provided an important clue that the variant was likely to be causal in the VEO-IBD case. The significance of this variant was subsequently confirmed by the identification of seven additional VEO-IBD cases that involve loss-of-function variants in the same gene, and by accompanying functional studies (Ouahed et al. manuscript in preparation).

The identification of additional unrelated patients with overlapping phenotypes and deleterious intragenic variants in the same gene constitutes a powerful strategy to establish the causality of a given candidate disease gene of uncertain clinical significance.^23^ To ascertain additional cases, we make extensive use of Matchmaker Exchange,^21^ as well as of ad hoc inquiries to other investigators who are actively working on phenotypically related conditions. In the case of the patient with VEO-IBD and bilateral hearing loss described above, additional unrelated cases with deleterious variants in the same gene were identified using Matchmaker Exchange and inquiries to external investigators including the VEO-IBD consortium (www.veoibd.org). Indeed, in this specific case, engagement across multiple institutions resulted in a fruitful collaboration with BGM. This work ultimately led to a novel disease gene discovery and diagnosis for multiple families (Ouahed, Snapper et al. manuscript in preparation).

In a second illustrative example (Table 1, case 5), the proband in the case presented to an emergency room with painful, dusky ischemic toes of uncertain etiology 3 days after the onset of a mild upper respiratory infection. Despite extensive diagnostic testing, consultants in rheumatology, vascular medicine, cardiology, and nephrology could not establish a diagnosis. The patient was therefore managed with a cocktail of drugs including glucocorticoids, calcium channel blockers, and anti-coagulants, and eventually recovered over many weeks. An astute clinician determined that other family members had presented with a range of similar findings (also in the setting of presumed infection), which in the most extreme case required amputation of affected digits. The family history was consistent with an autosomal dominant mode of inheritance, and WGS was used to analyze DNA both from the proband and an affected cousin. The genomic analysis for these two individuals identified 9 shared heterozygous variants and was posted on the crowdsourcing portal. A developmental geneticist highlighted a missense variant in Complement Factor 3 (C3), which alters a cleavage site required for production of C3 fragments that act to restrain excessive complement activation. Based on identification of a putative causative variant, follow-up testing of the proband revealed baseline elevated serum complement levels. While atypical hemolytic uremic syndrome (aHUS) was clinically ruled out in part due to the absence of acute renal failure, multiple family members carrying the familial C3 variant were subsequently found to exhibit microscopic hematuria. We posit that an infectious trigger for complement activation, superimposed upon a C3 variant that disrupts normal complement inactivation, contributes to vascular damage in this family. Given the suspected pathophysiology of the disorder, we propose that the C5 inhibitor Eculizumab, which is already clinically approved for the treatment of paroxysmal nocturnal hemoglobinuria and aHUS, should be discussed as a possible therapy during future episodes in affected family members. Thus, this case illustrates how an environmental trigger, notably infection, can interact with a specific genetic variant to cause disease. It also illustrates how identification of the causative gene may suggest therapeutic approaches that are otherwise not be considered in the care of affected family members.

In some cases, collaborators may also serve as matchmakers, by introducing additional related but independent cases. For example, in our investigation of the case of DA5 that involved musculoskeletal contractures and severe restrictive lung disease, an experimental collaborator whom we had contacted connected us with Norwegian clinicians who had identified a second intragenic variant in the *PIEZO2* gene in an independent case, thus establishing causality. This led to a multi-institutional discovery of *PIEZO2* as the cause of DA5.^23^

## Reporting evidence to referring clinicians

When sufficient evidence supports a causal relationship between a genetic variant and a disease phenotype, our group discusses the results with the patient’s referring physician and other pertinent clinical consultants in the context of the regular weekly meeting. For cases in which empiric management previously provided little benefit, additional therapeutic interventions may also be discussed. A comprehensive written report of the findings and potential treatments suggested by the genetic mechanism is submitted to the referring physician. Variants identified in research-based analyses are independently confirmed in a CLIA laboratory in accordance with return of results process defined in our IRB-approved protocol. The referring physician invites the patient and other relevant family members to visit the clinic for an on-site discussion of the results and for counseling, typically with a medical geneticist and/or a genetic counselor. This formal return of results provides a satisfying culmination to the genomic evaluation process, which is important since the overall process for each case often takes many months. The turnaround time for cases with novel gene discovery is 6 to 18 months, where the rate limiting steps are (1) the experimental work required to elucidate the function of the candidate gene (when it is unknown), and the functional impact of the candidate variant; and (2) the identification of independent cases with similar phenotypes caused by variants in the same gene.

## Completed projects and clinical significance

The main distinction between BGM and other programs aimed at clinical diagnosis of suspected monogenic disease is that our main focus is on discovering novel disease–gene associations, most typically in undiagnosed disease states. For this reason, cases with recognizable or known phenotypes are either directed elsewhere or are initially screened for the extant disease-causing variants linked to those phenotypes. Therefore, the majority of cases that come to the attention of the program described here are non-routine, high-complexity cases, and often referred from other programs, where they were deemed intractable.

To date, we have enrolled 244 families, sequenced DNA from 122 patients, along with additional samples from informative family members, analyzed 106 of these cases (Supp. Table 1), and elucidated a genetic etiology for 30 cases (Table 1). Another 48 cases (not listed here) have been resolved to the level of a potentially pathologic variant in one or a very small number of candidate genes, but require identification of additional related cases or functional experiments to demonstrate causality. Of the 30 cases solved by our program and listed in Table 1, 6 were referred to us with prior negative WES or gene panel sequencing, while at least 10 of the solved cases, or one third, afforded a genetic etiology for a previously unknown medical condition (Table 1). In addition, several of the families that were solved by this program had only one affected proband, and therefore required a more complicated disease gene discovery analysis than would otherwise be the case. Since our program’s primary focus is on novel disease–gene discovery, and since cases for which a known genetic variant is strongly suspected in advance of WES/WGS are excluded from our pipeline, it is notable that our ~28% (30/106) rate of gene identification is nominally equivalent to the 17–25% diagnostic yield reported by clinical genome sequencing programs.^24–28^

Additional examples of significant disease-associated gene identifications, summarized in Table 1, further illustrate specific features of the program. These cases include *PIEZO2* (a subtype of distal arthrogryposis, a musculoskeletal contracture and respiratory disease),^23^ *LOX* (familial aortic dissection),^29^ *WISP3* (precocious arthritis),^30^ *CHST11* (T cell lymphoma with limb abnormalities),^31^ complement factor *C3* gene (Chopra et al., manuscript in preparation), several cases of undiagnosed craniofacial dysmorphoses, and a gene that causes a rare form of lung cancer (Frank et al., manuscript in preparation) among others.

Table 1 also depicts a ratio of 21 WES to 9 WGS cases under “Sequencing strategy,” which is consistent with the ratio for all 122 patients, which includes 91 analyzed by WES and 43 by WGS. A total of 251 samples were submitted for WES, or ~2.8 samples per case, while 108 samples were submitted for WGS, or ~2.5 samples per case. While the majority of solved cases identify non-synonymous variants that should be captured by WES, the advantages of WGS include more uniform coverage, precise detection of regions of identity by descent, genome-wide data to call structural variants, and the opportunity to call potential non-coding regulatory variants within linkage disequilibrium peaks around disease-associated genes. For example, in a case with craniofacial anomalies and hypotonia we detected a translocation that disrupts intron 2 of *CAPZB* (Table 1, case 12). Also, in a novel syndrome of skeletal malformation and malignant lymphoproliferative disease (Table 1, case 1), a partial deletion of *CHST11* was detected which might have been difficult to detect with WES capture methodology. Finally, a progressive pseudorheumatoid arthropathy of childhood (PPAC) case (Table 1, case 2) benefited from careful detection of autozygous segments by WGS, which reduced the analysis to only 6% of the genome given the consanguineous family structure.

Some of our cases were diagnosed by identification of a novel variant in a known disease-associated gene, despite our selection criteria for gene discovery. Several reasons account for this circumstance. First, some referred disorders are misdiagnosed or undiagnosed simply because they are relatively rare and have not been previously seen by the referring clinical team. An example of such a case is PPAC, with a *WISP3* variant (Table 1, case 2). Second, some cases have had prior research grade WES and novel variants in known genes have been missed by the referring research team due to limitations of available WES interpretation tools. For example, this was the case in infantile-onset IBD with a variant in the disease-causing gene^32,33^ *DOCK8* (Table 1, case 4). Lastly, some patients have had WES or gene panels performed in clinical laboratories that do not follow-up with functional studies on reported variants of uncertain significance. This is illustrated by the *MED12* variant identified in a patient with congenital anomalies, polydactyly, and developmental delay. Thus, WES/WGS holds considerable value in rendering a definitive diagnosis in undiagnosed genetic cases. Even if deep re-analysis does not lead to the discovery of a novel disease-causing gene, clarification of the etiology of atypical, ambiguous, or challenging cases often expands our knowledge of the range of phenotypic states associated with specific genes and provides a definitive diagnosis for the patient and family.

In addition to providing insights into disease biology, the identification of disease-causing genes has broader medical ramifications. For example, once Mendelian disease-causing variants are validated through functional studies, the role of these genes in more common, related diseases states can be explored. For example, a newly discovered gene for Mendelian IBD may have immediate relevance to understanding the etiology of more common adult forms of IBD, such as Crohn’s disease or ulcerative colitis. As one approach to pursue such potential connections, gene-centric PheWAS^34,35^ statistical analysis can be performed across all phenotypic categories within available large-scale sequencing datasets. Such analyses can validate specific Mendelian disease gene findings in the context of common disease, and explore the broader value of the genetic information.

Other successful case analyses illustrate the potential of a modern genomic medicine service to provide insight into disease-causing pathways of direct therapeutic importance. For example, a 35-year-old Caucasian male presented to the hospital for evaluation of a personal and family history of thoracic aortic aneurysm and dissection (TAAD). He had undergone surgical repair of pectus excavatum at 2 years of age and was diagnosed with a large ascending aortic aneurysm at age 19. Based on these anomalies and other physical features including tall stature, high arched palate, and dental crowding, as well as a family history consistent with an autosomal dominant mode of inheritance, he was diagnosed of Marfan syndrome. However, genetic testing identified no variants in *FBN1*, *TGFBR1*, and *TGFBR2*, the genes that are associated with Marfan syndrome. Genetic testing performed in the proband’s affected mother also failed to reveal any causal variants in a larger collection of genes associated with connective tissue disorders: *ACTA2*, *COL3A1*, *MYH11*, *SLC2A10*, *SMAD3*, or *MYLK*. Therefore, WGS was performed for the proband and his affected cousin and identified a p.Met298Arg missense variant in the elastin and collagen cross-linking enzyme, Lysyl oxidase, encoded by the *LOX* gene.^29^ This variant would place a repulsive positive charge from Arg in the Cu^2+^ binding active site of the enzyme. Lysyl oxidase activity in skin declines with low dietary Cu^2+^ and increases with repletion.^36^ Taken together, these findings suggest the hypothesis that Cu^2+^ supplementation, within US Department of Agriculture (USDA) safety guidelines, might augment the Cu^2+^-dependent enzymatic function of LOX and therefore help protect against aortic disease in individuals carrying this variant. This hypothesis is now directly testable in a murine model where the specific genetic variant has been knocked-in to the endogenous *Lox* locus. In addition, family members carrying the p.Met298Arg variant are now being serially monitored by echocardiography and by magnetic resonance angiography for aortic dilatation, which may prevent significant morbidity and mortality when identified early in the course of disease rather than at the time of rupture.

## Challenges

Despite the successes mentioned above, programs for integrated genomic medicine, such as that described here, face a number of challenges. These include the time required to accumulate evidence for disease causality of candidate genes and variants, the development of a standardized clinical classification guideline for variants of uncertain significance, gaining acceptance of clinical reimbursement standards among third-party payers, and assessing and reporting incidental findings. For example, in addition to the 30 solved cases reported in Table 1, another 48 cases have been resolved to the level of single or few strong candidate genes, but require further work to identify additional phenotypically related cases with intragenic variants or to perform the requisite functional experiments needed to establish causality. Such experimental work, which has the potential to greatly strengthen the genotype–phenotype correlation, is not currently a reimbursable component of the clinical assessment. Thus, a significant time lag in case resolution and a substantial funding shortfall are to be expected in any genomic medicine program that emphasizes new gene discovery in undiagnosed cases over routine genetic diagnosis.

In addition, while the cost of clinical WES/WGS has dramatically declined in recent years, the reimbursement policies of health insurance companies and payers remain heavily biased to single gene tests and gene panels. Though insurance companies are starting to recognize the significant clinical utility of genomic sequencing, initiatives such as ours have been funded by institutional and extramural research sources. Ultimately, we envision that the secondary analysis of genomic sequencing and the integrated clinical practice of genomic medicine will be supported by third-party payers. Currently, the research components of such programs continue to depend upon institutional and some targeted extramural support. Ultimately, the funding of patient-level experimental biology may be reflected in the reimbursement for clinical care, much as therapeutic drug trials are currently cross funded by payers in oncology. To this end, we plan to leverage the billing expertise of the Brigham and Women’s Hospital Departments of Medicine (for professional billing) and Pathology (for technical billing), while implementing the new revenue streams available under the revised coding structure for genetic testing. In January 2015, the American Medical Association (AMA) released revised Current Procedural Terminology (CPT) codes that reflect recent advances in genomic medicine, including WES/WGS and the re-evaluation of existing sequence data for both patients and family members.^37^ We have begun to prepare to utilize these changes to CPT coding and billing to maximize the clinical reimbursement for WES/WGS. Recognition of the clinical utility of WES/WGS by clinical and translational research programs will hopefully provide payers with a logical framework in which to evaluate evidence for the value of genomic medicine to the health care system.

## Conclusion

Recent advances in genomic sequencing have enabled the resolution of undiagnosed cases of human disease and can provide answers to families and clinicians. The model described here is based on well-defined genetic entry points into human biology that lead to disease gene discovery, with both clinical implications and subsequent mechanistic evaluation. This model integrates state-of-the-art genetic research with innovation across multiple domains, including a refined computational analysis pipeline, translatable functional assays, improved clinical phenotyping, and therapeutic intervention. As this program effectively assimilates a multidisciplinary team of clinical and research expertise in the task of diagnosing and treating these debilitating conditions, this integrated monogenic disease gene discovery model can be readily adapted by other academic medical centers. Lastly, by promoting the clinical implementation of genomic medicine, the genomic medicine program described here also affords a unique educational opportunity for trainees and investigators across many disciplines of biomedicine.

### Data availability

Additional data supporting the findings of this study are available within the supplementary information files. The genomic datasets generated and/or analyzed during the current study are not publicly available due to data and privacy protection considerations but may be available on justified request.

## Electronic supplementary material

Supplementary Information

## Electronic supplementary material

**Supplementary information** accompanies the paper on the *npj Genomic Medicine* website (10.1038/s41525-018-0060-9).
