# Supplementary material for: An integrated clinical program and crowdsourcing strategy for genomic sequencing and Mendelian disease gene discovery
Source: NPJ Genom Med. 2018 Aug 13;3:21. doi: 10.1038/s41525-018-0060-9 (PMC6089983; doi:10.1038/s41525-018-0060-9)
Supplement: Supplementary file 1 — Supplementary Information [file 41525_2018_60_MOESM1_ESM.docx]

**An Integrated Clinical Program and Crowdsourcing Strategy for Genomic Sequencing**

**and Mendelian Disease Gene Discovery**

Alireza Haghighi^1,2,3,4,5^, Joel B. Krier^1^, Agnes Toth-Petroczy^1^, Christopher A. Cassa^1,5^, Natasha Y. Frank^1,5^, Nikkola Carmichael^1^, Elizabeth Fieg^1^, Andrew Bjonnes^1^, Anwoy Mohanty^1^, Lauren C. Briere^6^, Sharyn Lincoln^7^, Stephanie Lucia^7^, Vandana A. Gupta^1^, Onuralp Söylemez^1^, Sheila Sutti^1^, Kameron Kooshesh^1^, Haiyan Qiu^1^, Christopher J. Fay^1^, Victoria Perroni^1^, Jamie Valerius^1^, Meredith Hanna^1^, Alexander Frank^1^, Jodie Ouahed^8^, Scott B. Snapper^8, 19^, Angeliki Pantazi^1^, Sameer S. Chopra^18^ , Ignaty Leshchiner^5^, Nathan O. Stitziel^20^ , Anna Feldweg^10^, Michael Mannstadt^9^, Joseph Loscalzo^10^, David A. Sweetser^6^, Eric Liao^11^, Joan M. Stoler^7^, Catherine B. Nowak^7,12^, Pedro A. Sanchez-Lara^13^, Ophir D. Klein^14^, Hazel Perry^14^, Nikolaos A. Patsopoulos^1,5,15^, Soumya Raychaudhuri^1,5,16,17^, Wolfram Goessling^1,5,18,19^, Undiagnosed Diseases Network, Brigham and Women’s Hospital FaceBase Project, Brigham Genomic Medicine (BGM), Robert C. Green^1,5^, Christine E. Seidman^2,3,4,5^, Calum A. MacRae^1,2,5^, Shamil R. Sunyaev^1,5^, Richard L. Maas^1^, and Dana Vuzman^1,2,5^

^1^ Division of Genetics, Department of Medicine, Brigham and Women’s Hospital, Harvard Medical School, Boston, MA 02115, USA

^2^ Division of Cardiovascular Medicine, Department of Medicine, Brigham and Women’s Hospital, Harvard Medical School, Boston, MA 02115, USA

^3^ Department of Genetics, Harvard Medical School, Boston, MA 02115, USA

^4^ Howard Hughes Medical Institute, Brigham and Women’s Hospital, Boston, MA 02115 USA

^5^ Broad Institute of Harvard and MIT, Cambridge, MA 02142, USA

^6^ Division of Medical Genetics and Metabolism, Department of Pediatrics, Massachusetts General Hospital, Harvard Medical School, Boston, MA 02114, USA

^7^ Division of Genetics and Genomics, Department of Medicine, Boston Children’s Hospital, Harvard Medical School, Boston, MA 02115, USA

^8^ Division of Gastroenterology, Hepatology and Nutrition, Department of Medicine, Boston Children's Hospital, Harvard Medical School, Boston, MA 02115, USA

^9^ Endocrine Unit, Massachusetts General Hospital and Harvard Medical School, Harvard Medical School, Boston, MA 02114, USA.

^10^ Department of Medicine, Brigham and Women's Hospital, Harvard Medical School, Boston, MA 02215, USA

^11^ Division of Plastic and Reconstructive Surgery, Department of Surgery, Massachusetts General Hospital, Harvard Medical School, Boston, MA 02114, USA

^12^ Feingold Center for Children, Children’s Hospital Boston at Waltham, Waltham, MA 02453, USA

^13^ Department of Pediatrics, Cedars-Sinai Medical Center, David Geffen School of Medicine at UCLA, Los Angeles, CA, 90048, USA

^14^ Department of Orofacial Sciences, University of California San Francisco, San Francisco, CA 94143, USA

^15^ Dept. of Neurology, Brigham and Women’s Hospital, Harvard Medical School, Boston, MA 02115, USA

^16^ Division of Rheumatology, Allergy and Immunology, Department of Medicine, Brigham and Women’s Hospital and Harvard Medical School, Boston, MA 02115, USA

^17^ Institute of Inflammation and Repair, University of Manchester, Manchester, UK

^18^ Dana-Farber Cancer Institute, Boston, MA 02115, USA

^19^ Division of Gastroenterology, Hepatology and Endoscopy, Department of Medicine, Brigham and Women's Hospital, Harvard Medical School, Boston, MA 02115, USA

20 Cardiovascular Division, Department of Medicine; Department of Genetics; McDonnell Genome Institute,

Washington University School of Medicine, St Louis MO, 63110, USA

**Running title:** A program for clinical disease gene discovery

**Correspondence:** Dana Vuzman (dvuzman@broadinstitute.org) and Alireza Haghighi (haghighi@genetics.med.harvard.edu), Division of Genetics, Brigham and Women’s Hospital, Harvard Medical School, New Research Building, 77 Avenue Louis Pasteur, Boston, MA 02115

**Supplementary Data**

WES is performed by a CLIA or quality research sequene provider; WGS is performed by the Illumina Clinical Services Laboratory (Illumina, Inc; San Diego, CA). Briefly, genomic DNA is randomly fragmented and then sequenced using 100 nucleotide paired-end reads on an Illumina HiSeq 2000 Sequencer at an average depth of 30x coverage for whole genome sequences and 50-100x for whole exome sequences^1^. The resulting BAM files are converted to FASTQ format and realigned to the human reference sequence [University of California Santa Cruz (UCSC) hg19/GRCh37 assembly] using the Burroughs-Wheeler Alignment tool (BWA-MEM) ^2,3^ in paired-end mode. Duplicated reads resulting from PCR over-amplification or optical duplication are flagged and discarded by the MarkDuplicates module of Picard tools version 2.5 (<http://picard.sourceforge.net/>). Targeted local realignment is then performed near known short insertions and deletions (indels) and by base quality recalibration using the Genome Analysis Toolkit (GATK, version v3.7) ^4,5^. Single nucleotide variants (SNVs) and indels are discovered using batch calling with a set of unrelated individuals by using the HaplotypeCaller and GenotypeGVCFs modules in GATK. Variants are prioritized using in-house developed tools depending on the inheritance mode suspected in any particular case. The models used for this purpose take into account population genetics parameters as well as technical artifacts observed in unrelated samples and also statistics from databases such as the Genome Aggregation Database (gnomAD) and 1000 genomes. The functional consequence of each variant is predicted using Variant Effect Predictor (Release 85) ^6^ . Structural variants (SV) are identified by comparing the average coverage for control samples to the proband sample over the same exons. This is performed with GenomeSTRiP^7^ for whole genome samples and with xHMM^8^ for whole exome samples. Sample quality control is assessed by calculating coverage over exons; assuring the sex of the sample, calculated by comparing the ratio of the Y chromosome coverage relative to the entire genome, matches clinical records; comparing observed autozygosity to expected values using PLINK^9^ for WGS samples; comparing metrics (including het/hom ratio, TiTv ratio, number of variants) from GATK’s VariantEval against unrelated high quality sequences; ensuring counts of synonymous, missense, frameshift, inframe and splice-site variants are within expected ranges; and assessing ancestry with principal component analysis using HGDP continental populations as a reference^10,11^ (<http://www.hagsc.org/hgdp/>). Additionally, any variants labeled pathogenic or likely pathogenic in either ClinVar^12^ (<http://www.ncbi.nlm.nih.gov/clinvar>) or HGMD^13^ (<http://www.hgmd.org>) with allele frequency less than 2.5% are reported. Variants not found in 1000 Genomes^14^, in dbSNP (Single Nucleotide Polymorphism Database) build 147^15^, or in the Genome Aggregation Database (gnomAD, with 123,136 exomes and 15,496 genomes) dataset at a frequency below 0.1% are generally considered for analysis in recessive cases, while higher allele frequencies are considered in dominant cases where the prevalence of disease may be higher^16^. Homozygous and compound heterozygous sequence variants are evaluated in putative recessive cases and heterozygous variants are also considered in dominant cases.

**Supplementary Table 1: Summary of cases**

|  | **Status** | **#Cases** |
| --- | --- | --- |
| Completed Projects | Published ^17-26^ (Table 1) | 19 |
|  | In prep./Submitted (Table 1) | 4 |
|  | Experimental validation (Table 1) | 5 |
|  | Solved & closed without publication (Table 1)  Closed without solution (Data not shown) | 2  42 |
| Pending projects | Candidate gene identified (Data not shown) | 48 |
|  | Analysis in progress (Data not shown) | 25 |
|  | Pending sequencing (Data not shown) | 104 |
|  | **Total** | **244** |

**References**

1 Bentley, D. R. *et al.* Accurate whole human genome sequencing using reversible terminator chemistry. *Nature* **456**, 53-59, doi:10.1038/nature07517 (2008).

2 Li, H. & Durbin, R. Fast and accurate short read alignment with Burrows-Wheeler transform. *Bioinformatics* **25**, 1754-1760, doi:10.1093/bioinformatics/btp324 (2009).

3 Li, H. Exploring single-sample SNP and INDEL calling with whole-genome de novo assembly. *Bioinformatics* **28**, 1838-1844, doi:10.1093/bioinformatics/bts280 (2012).

4 DePristo, M. A. *et al.* A framework for variation discovery and genotyping using next-generation DNA sequencing data. *Nature genetics* **43**, 491-498, doi:10.1038/ng.806 (2011).

5 McKenna, A. *et al.* The Genome Analysis Toolkit: a MapReduce framework for analyzing next-generation DNA sequencing data. *Genome Res* **20**, 1297-1303, doi:10.1101/gr.107524.110 (2010).

6 Yourshaw, M., Taylor, S. P., Rao, A. R., Martin, M. G. & Nelson, S. F. Rich annotation of DNA sequencing variants by leveraging the Ensembl Variant Effect Predictor with plugins. *Brief Bioinform* **16**, 255-264, doi:10.1093/bib/bbu008 (2015).

7 Handsaker, R. E., Korn, J. M., Nemesh, J. & McCarroll, S. A. Discovery and genotyping of genome structural polymorphism by sequencing on a population scale. *Nature genetics* **43**, 269-276, doi:10.1038/ng.768 (2011).

8 Fromer, M. *et al.* Discovery and statistical genotyping of copy-number variation from whole-exome sequencing depth. *Am J Hum Genet* **91**, 597-607, doi:10.1016/j.ajhg.2012.08.005 (2012).

9 Purcell, S. *et al.* PLINK: a tool set for whole-genome association and population-based linkage analyses. *Am J Hum Genet* **81**, 559-575, doi:10.1086/519795 (2007).

10 Wang, C. *et al.* Ancestry estimation and control of population stratification for sequence-based association studies. *Nature genetics* **46**, 409-415, doi:10.1038/ng.2924ng.2924 [pii] (2014).

11 Wang, C., Zhan, X., Liang, L., Abecasis, G. R. & Lin, X. Improved ancestry estimation for both genotyping and sequencing data using projection procrustes analysis and genotype imputation. *Am J Hum Genet* **96**, 926-937, doi:10.1016/j.ajhg.2015.04.018S0002-9297(15)00155-X [pii] (2015).

12 Landrum, M. J. *et al.* ClinVar: public archive of relationships among sequence variation and human phenotype. *Nucleic Acids Res* **42**, D980-985, doi:10.1093/nar/gkt1113 (2014).

13 Stenson, P. D. *et al.* Human Gene Mutation Database (HGMD): 2003 update. *Hum Mutat* **21**, 577-581, doi:10.1002/humu.10212 (2003).

14 Genomes Project, C. *et al.* A map of human genome variation from population-scale sequencing. *Nature* **467**, 1061-1073, doi:10.1038/nature09534 (2010).

15 Sherry, S. T. *et al.* dbSNP: the NCBI database of genetic variation. *Nucleic Acids Res* **29**, 308-311 (2001).

16 Lek, M. *et al.* Analysis of protein-coding genetic variation in 60,706 humans. *Nature* **536**, 285-291, doi:10.1038/nature19057 (2016).

17 Chopra, S. S. *et al.* Inherited CHST11/MIR3922 deletion is associated with a novel recessive syndrome presenting with skeletal malformation and malignant lymphoproliferative disease. *Molecular genetics & genomic medicine* **3**, 413-423, doi:10.1002/mgg3.152 (2015).

18 Cassa, C. A. *et al.* An argument for early genomic sequencing in atypical cases: a WISP3 variant leads to diagnosis of progressive pseudorheumatoid arthropathy of childhood. *Rheumatology* **55**, 586-589, doi:10.1093/rheumatology/kev367 (2016).

19 Lee, V. S. *et al.* Loss of function mutation in LOX causes thoracic aortic aneurysm and dissection in humans. *Proc Natl Acad Sci U S A* **113**, 8759-8764, doi:10.1073/pnas.1601442113 (2016).

20 Brownstein, C. A. *et al.* An international effort towards developing standards for best practices in analysis, interpretation and reporting of clinical genome sequencing results in the CLARITY Challenge. *Genome biology* **15**, R53, doi:10.1186/gb-2014-15-3-r53 (2014).

21 Coste, B. *et al.* Gain-of-function mutations in the mechanically activated ion channel PIEZO2 cause a subtype of Distal Arthrogryposis. *Proceedings of the National Academy of Sciences of the United States of America* **110**, 4667-4672, doi:10.1073/pnas.1221400110 (2013).

22 Zeissig, S. *et al.* Early-onset Crohn's disease and autoimmunity associated with a variant in CTLA-4. *Gut* **64**, 1889-1897, doi:10.1136/gutjnl-2014-308541 (2015).

23 Mukherjee, K. *et al.* Actin capping protein CAPZB regulates cell morphology, differentiation, and neural crest migration in craniofacial morphogenesisdagger. *Hum Mol Genet* **25**, 1255-1270, doi:10.1093/hmg/ddw006 (2016).

24 Faden, M. *et al.* Identification of a Recognizable Progressive Skeletal Dysplasia Caused by RSPRY1 Mutations. *Am J Hum Genet* **97**, 608-615, doi:10.1016/j.ajhg.2015.08.007 (2015).

25 NM_003188.3(MAP3K7):c.521G>A (p.Cys174Tyr), https://www.ncbi.nlm.nih.gov/clinvar/RCV000414538 (2016).

26 Kusumam, J. *et al.* Reply. *J Allery Clin Immunol* 139*,* 1720-1721, doi: http://dx.doi.org/10.1016/j.jaci.2016.12.983 (2017).
